# 48 Hour Hospice Home Immersions - AY 2018-19: Student Learning and Skills to Augment Their Career

**DOI:** 10.1093/geroni/igaf122.4051

**Published:** 2025-12-31

**Authors:** Tyler Dentry, Kevin Reale, Marilyn Gugliucci

**Affiliations:** University of New England College of Osteopathic Medicine, Biddeford, Maine, United States; University of New England College of Osteopathic Medicine, Biddeford, Maine, United States; University of New England College of Osteopathic Medicine, Kennebunk, Maine, United States

## Abstract

Medical student end-of-life care (EOL) and palliative medicine (PM) training has been cited as inadequate; creating gaps in student preparation and confidence in end-of-life care. To address this, UNECOM developed the Learning by Living: 48-hour Hospice Immersion in 2014, to create a unique immersive learning experience in EOL/PM care. The purpose of this analysis was to determine the changes in students’ perspectives before and after the immersion and what learning/skills they plan to apply as a physician. UNECOM student volunteers were immersed for 48 hours in a hospice house to conduct ethnographic research through three phases: (1) Pre-Field Work; (2) Field Work; and (3) Post-Field Work. Twelve student journals from the 2018-2019 academic year were analyzed in five steps: (1) selected student journals read to identify themes; (2) journals re-read notating and selecting quotes; (3) code book created with agreed upon theme definitions (inter-rater reliability); (4) data coded into identified themes; and (5) final analysis conducted using NVivo Qualitative Software. Data analyses resulted in three key themes that reflected knowledge, skills, and attitudes to augment students’ success in EOL: (1) preconceived notions of the hospice experience, (2) growth and development as a professional, and (3) person-centered care in the hospice environment. Quotes extracted from student journals highlighted impactful and unique experiences for developing medical students’ confidence to work with terminal patients. Impactful immersive education such as this 48-Hour Hospice Immersion provides EOL and PM education in a clinical setting that benefits students and their future patients.

